# A tRNA modification pattern that facilitates interpretation of the genetic code

**DOI:** 10.3389/fmicb.2024.1415100

**Published:** 2024-06-12

**Authors:** Isao Masuda, Ya-Ming Hou

**Affiliations:** Department of Biochemistry & Molecular Biology, Thomas Jefferson University, Philadelphia, PA, United States

**Keywords:** tRNA anticodon, position 34 of the anticodon, position 37 on the 3′-side of the anticodon, post-transcriptional modifications, modification circuitry

## Abstract

Interpretation of the genetic code from triplets of nucleotides to amino acids is fundamental to life. This interpretation is achieved by cellular tRNAs, each reading a triplet codon through its complementary anticodon (positions 34–36) while delivering the amino acid charged to its 3′-end. This amino acid is then incorporated into the growing polypeptide chain during protein synthesis on the ribosome. The quality and versatility of the interpretation is ensured not only by the codon-anticodon pairing, but also by the post-transcriptional modifications at positions 34 and 37 of each tRNA, corresponding to the wobble nucleotide at the first position of the anticodon and the nucleotide on the 3′-side of the anticodon, respectively. How each codon is read by the matching anticodon, and which modifications are required, cannot be readily predicted from the codon-anticodon pairing alone. Here we provide an easily accessible modification pattern that is integrated into the genetic code table. We focus on the Gram-negative bacterium *Escherichia coli* as a model, which is one of the few organisms whose entire set of tRNA modifications and modification genes is identified and mapped. This work provides an important reference tool that will facilitate research in protein synthesis, which is at the core of the cellular life.

## Introduction

**Figure 1:**
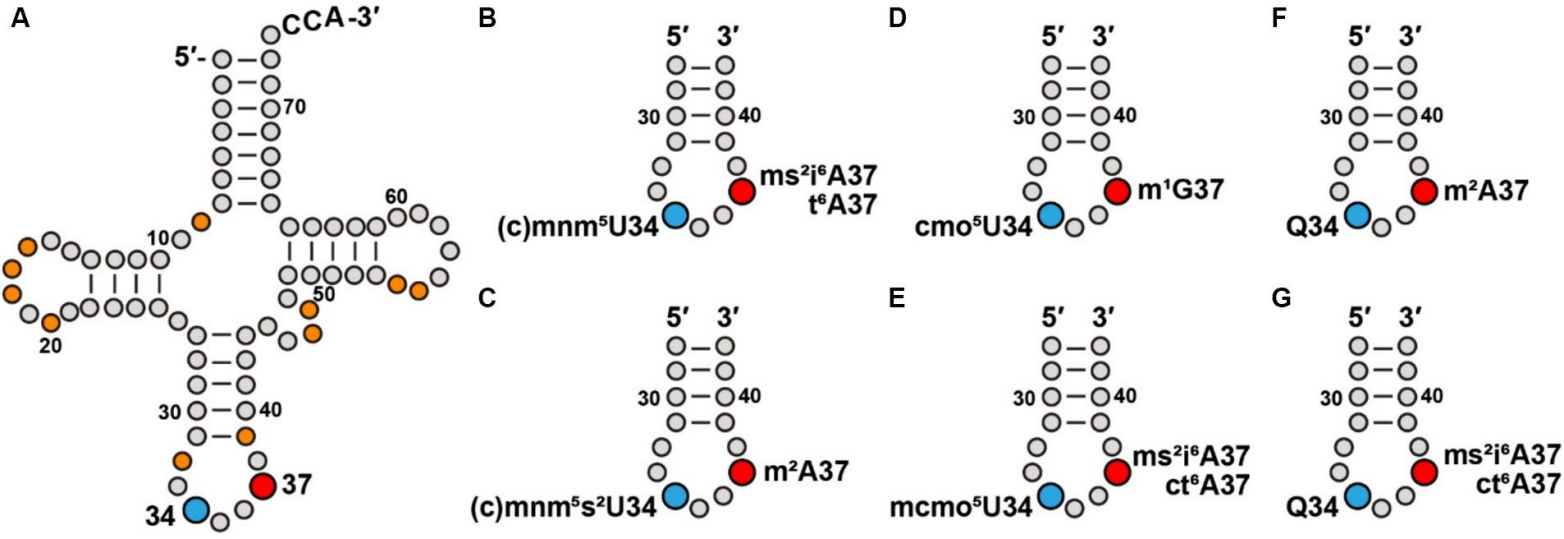
Modifications at tRNA positions 34 and 37 contribute to a modification pattern. **(A)** Modifications at positions 34 and 37 of *E. coli* tRNAs, in blue and red, respectively, in the cloverleaf structure that also include other commonly present modifications (in orange), such as s^4^U8, D16 (D: dihydrouridine), D17, G_m_18, D20, C_m_/U_m_32, ψ39 (ψ: pseudouridine), m^7^G46, acp^3^U47 (acp^3^U, 3-amino-3-carboxy-propyl-uridine), m^5^U54, and ψ55, most of which contribute to stabilization of the tRNA structure. **(B)** Association of cmnm^5^U34 and mnm^5^U34 with ms^2^i^6^A37 or t^6^A37 series in an ASL. **(C)** Association of cmnm^5^s^2^U34 and mnm^5^s^2^U34 with m^2^A37 in an ASL. **(D)** Association of cmo^5^U34 with m^1^G37 in an ASL. **(E)** Association of mcmo^5^U34 with ms^2^i^6^A37 or with ct^6^A37 in an ASL. **(F)** Association of Q34 with m^2^A37 in an ASL. **(G)** Association of Q34 with ms^2^i^6^A37 or with ct^6^A37 in an ASL.

The genetic code is universally conserved, with minor exceptions, as the central mechanism that translates genetic information stored in nucleic acids to amino acid sequences of proteins. This code consists of 64 triplets of nucleotides, of which 61 are sense codons, each specifying the insertion of an amino acid to protein synthesis, while the remaining 3 are non-sense codons, each signaling termination of protein synthesis. Importantly, interpretation of the genetic code is entirely dependent on cellular tRNAs providing matching anticodons to each codon, while delivering the specified amino acid as the building block to protein synthesis. The speed and quality of interpreting each codon is essential for life. This demand is addressed by post-transcriptional modifications in tRNA molecules, most notably at position 34 and at position 37 (Figure 1A). The presence of tRNA modifications at positions 34 and 37 also controls and regulates the codon-anticodon wobble pairing (Agris et al., 2018), thus solving the conundrum that organisms across the tree of life usually have a fewer number of tRNA anticodons relative to 64 codons of the genetic code. Notably, while all native tRNA molecules contain an extensive set of modifications, those at positions 34 and 37 encompass the greatest structural diversity. Some of these modifications are also functionally coupled to tRNA aminoacylation and to maintenance of the translational reading frame (Bjork et al., 1987; Muramatsu et al., 1988; Gamper et al., 2015; Thiaville et al., 2016; Gamper et al., 2021a,b; Masuda et al., 2022). Additionally, tRNA modifications at these two positions can be inducible, responding to cellular stress to facilitate translation of codon-biased stress genes (Endres et al., 2015). Indeed, dysregulation of tRNA modifications at these two positions is linked to various pathologies (Suzuki, 2021). However, despite the importance, how each anticodon is associated with a modification at positions 34 and/or 37 is not obvious from the genetic code and is not readily predictable, as subtle changes of the modification can alter the codon-anticodon pairing. This lack of information has limited researchers from a better understanding of the genetic code. Here, we address this critical gap, by revealing a pattern of tRNA modifications that associates the modifications at positions 34 and 37 of each tRNA to translation of its codon(s). We use this pattern to convey the information of modifications in a systematic organization that can be referenced by users. Notably, while the genetic code is conserved in evolution, the pattern of modifications is specific to each organism, depending on its GC content, codon usage, and dynamics of the tRNA repertoires (Diwan and Agashe, 2018).

We provide the pattern of tRNA modifications for *E. coli* strain MG1655 – one of the few organisms whose entire set of tRNA modifications is known and confirmed by identification of the associated modification enzyme(s) of each (de Crecy-Lagard and Jaroch, 2021). While some modifications are synthesized by one enzyme, others are synthesized in a pathway involving several enzymes. An example of the former is the *N*^1^-methylation of guanosine at position 37 (m^1^G37) by the TrmD tRNA methyltransferase (Hou et al., 2017), while an example of the latter is cmo^5^U34 (see the abbreviation below), which requires four enzymes catalyzing the successive conversion of U34 to ho^5^U34 and then to cmo^5^U34 (Sakai et al., 2019). The development of the pattern of modifications of *E. coli* is an important step to better understand the relationship between each codon and its anticodons for accurate interpretation of the genetic code. It also lays the framework for developing the pattern for related organisms and even higher eukaryotes, as the chemical structure of some modifications at position 34 or 37 are conserved (e.g., I34, m^1^G37, and ms^2^i^6^A37) or were evolved to a similar structure by addition of a new chemical moiety (e.g., from t^6^A37 to ms^2^t^6^A37).

Importantly, given the power of the genetic tools available to *E. coli*, investigation of each modification in the pattern is feasible, even if it is essential for cell viability (Masuda et al., 2019; Masuda and Hou, 2023). Additionally, *E. coli* is the single model organism that has received substantial advancement of bioengineering to expand the genetic code. Notably, while the genetic code provides the basic framework to synthesize proteins with the canonical 20 amino acids and selenocysteine (see below), efforts are being made to expand this code to incorporate a non-canonical amino acid at a site-specific position to diversify the structure and function of proteins, thus paving the way to protein-based medicines and industrial biocatalysts. Active efforts are aimed to engineer a tRNA to read a non-sense codon or a quadruplet codon for site-specific delivery of a non-canonical amino acid to protein synthesis (Shandell et al., 2021; Gamper et al., 2022; Kim et al., 2024). These efforts inevitably must consider tRNA modifications at positions 34 and 37 to control the quality of the engineered codon-anticodon pairing. For example, in a genetically isolated derivative of *E. coli* Pro(GGG) tRNA (see abbreviations below) that would be a strong candidate for reading of a quadruplet codon motif such as CCC-C, we show that the presence of m^1^G37, which disrupts the Watson-Crick (W-C) base pairing of the guanosine, readily compromises this activity (Gamper et al., 2015, 2021a,b), pointing to the need to better engineer this tRNA for genetic code expansion. Thus, the tRNA modification pattern as shown here will serve as a critical reference for consideration to push the frontier forward of genetic code expansion.

## Abbreviations in the pattern of *E. coli* modifications

To date, about 150 different types of modifications have been identified in all classes of RNAs, including tRNAs, mRNA, rRNAs, and other RNAs (Boccaletto et al., 2018). Focusing on tRNAs specifically, we analyzed the entire set of 47 unique sequences of *E. coli* MG1655 and compiled all the modifications found at positions 34 and 37 (Bjork and Hagervall, 2014), followed by literature searches to update the information (Keseler et al., 2021). We developed the tRNA modification pattern by showing each codon of the genetic code next to its anticodon(s), and by showing each anticodon with the modifications at positions 34 and 37. Modifications at position 34 include: mnm^5^U (5-methyl-amino-methyl-uridine), cmnm^5^U_m_ (5-carboxy-methyl-aminomethyl-2′-*O*-methyl uridine), C_m_ (2′-*O*-methyl cytidine), cmo^5^U (uridine 5-oxyacetic acid), ac^4^C (*N*^4^-acetyl-cytidine), mcmo^5^U (5-(methoxy)-carbonyl methoxy-uridine), mcmo^5^U_m_ (5-(methoxy)-carbonyl methoxy-2′-*O*-methyl-uridine), cmnm^5^s^2^U (5-carboxy-methyl-amino-methyl-2-thiouridine), mnm^5^s^2^U (5-methyl-amino-methyl-2-thiouridine), I (inosine), L (lysidine), Q (queuosine), and gluQ (glutamyl-queuosine). Conversely, modifications at position 37 include: ms^2^i^6^A (2-methylthio-*N*^6^-isopentenyl-adenosine), i^6^A (*N*^6^-isopentenyl-adenosine), m^1^G (*N*^1^-methyl-guanosine), m^2^A (2-methyl-adenosine), ct^6^A (cyclic *N*^6^-threonyl-carbamoyl-adenosine), m^6^t^6^A (*N*^6^-methyl-*N*^6^-threonyl-carbamoyl-adenosine), and m^6^A (*N*^6^-methyl-adenosine). Below we describe each modified nucleotide by the abbreviated form and follow it with the number 34 or 37, indicating its position in the tRNA sequence framework. We also cite each tRNA by the three-letter code of the amino-acid identity, followed by the modified anticodon in parentheses. Thus, Arg(ICG) means that it is tRNA^Arg^ with the anticodon that is modified from ACG to ICG in the native tRNA.

## The modification pattern at position 34 of the tRNA wobble nucleotide

**Figure 2:**
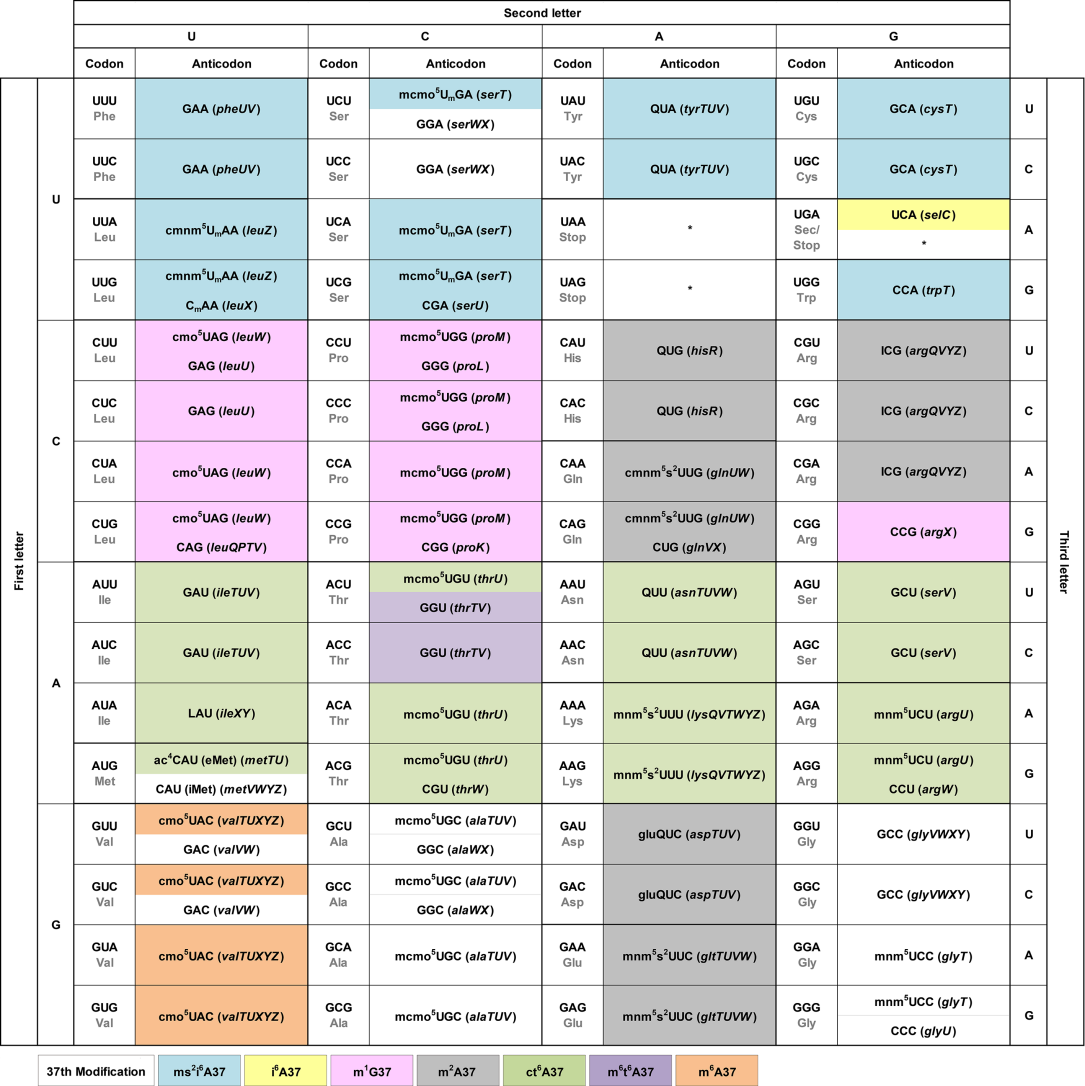
The modification pattern of *E. coli* tRNAs. Each sense codon of the genetic code is shown with its anticodon(s) that can form W-C or wobble base pairs. Each anticodon is shown with the identified modifications at position 34 directly in the genetic code table, while the modifications at position 37 shown by coloring. The associated tRNA gene that expresses each anticodon is shown in italic in parentheses. Each non-sense codon is marked with a * symbol.

We integrate the tRNA modification pattern into the genetic code table to provide an easy access to the association of each codon with the matching anticodon(s) (Figure 2). At each codon, we show directly in the table the modification at position 34 of the anticodon, which is responsible for wobble base pairing with the codon. We include the tRNA gene that expresses each anticodon in parentheses to facilitate cross-referencing with published work. Although specific patterns of modifications at position 34 were reported previously for all three domains of life (Grosjean et al., 2010), we describe below a few trends for bacterial tRNAs.

U34 is consistently modified to one of two series. The cmnm^5^U34 series includes cmnm^5^U_m_34 and mnm^5^s^2^U34 [e.g., Arg(mnm^5^UCU), Gln(cmnm^5^s^2^UUG), Glu(mnm^5^s^2^UUC), Gly(mnm^5^UCC), Leu(cmnm^5^U_m_AA), and Lys(mnm^5^s^2^UUU)], while the cmo^5^U34 series includes mcmo^5^U34 [e.g., Ala(mcmo^5^UGC), Pro(mcmo^5^UGG), Thr(mcmo^5^UGU), and Ser(mcmo^5^U_m_GA)]. These two series share the common 5-carboxyl group modification of uridine. The only exception without any modification of U34 is in the anticodon of Sec(UCA) (Sec: selenocysteine), which inserts Sec to an internal UGA codon in mRNAs using a different decoding mechanism that specifies the site of insertion by a 3′-stem-loop structure and requires dedicated accessory factors (Commans and Bock, 1999). This U34, however, is modified in human Sec(mcm^5^UCA) (Songe-Moller et al., 2010). By contrast, the modification status of C34 is more diverse, which can be unmodified as in Arg(CCG), Arg(CCU), Gln(CUG), Gly(CCC), Leu(CAG), iMet(CAU) (the initiator tRNA^Met^), Pro(CGG), Thr(CGU), and Trp(CCA), or modified as in Leu(C_m_AA) or in eMet(ac^4^CAU) (the elongator tRNA^Met^). An important modification is L34 in Ile(LAU), which is the single determinant that reads the Ile codon AUA and discriminates against the Met codon AUG, while also serving as the major determinant that prevents the tRNA from mis-aminoacylation with Met (Muramatsu et al., 1988; Suzuki and Miyauchi, 2010). Similarly, A34 is consistently modified to I34, without exception, which expands the capacity of I34 to base-pair with A/C/U. This A-to-I modification is found in the single tRNA Arg(ICG). In contrast, G34 is mostly unmodified, except when it is in the GUX sequence, where it is modified to Q34 as in Tyr(QUA), His(QUG), Asn(QUU), and in Asp(gluQUC). The modification to Q34 equalizes the quality of base pairing with C/U (Morris et al., 1999), thus conferring a similar strength and efficiency of decoding of the two related codons.

## The modification pattern at position 37 on the 3′-side of the tRNA anticodon

We show the modification at position 37 on the 3′-side of each anticodon using different colors (Figure 2). The nucleotide at position 37 is predominantly a purine, whose modification can provide a stacking interaction with the nucleotide at position 36 of the anticodon to stabilize the structure of the anticodon-stem loop (ASL) (Konevega et al., 2004). This stabilization is necessary to neutralize differences of the ASL sequence for docking into the ribosome decoding site with a uniform speed and quality during protein synthesis (Konevega et al., 2004; Seelam Prabhakar et al., 2021). It is also necessary to assist the extensive conformational rearrangement of the ASL during the ribosome translocation to move down to the next codon (Nguyen et al., 2019). Indeed, deficiency of the modification at position 37 can cause ribosome stalling and +1-frameshifting (Masuda et al., 2021), indicating the importance of the modification in translation of the genetic code. Analysis of the modification pattern at position 37 in relationship with the nucleotide at position 36 identifies the following trends.

With U36-ending anticodons, the modification at position 37 is mostly ct^6^A37 and in one case it is m^6^t^6^A37 [in Thr(GGU)]. With C36-ending anticodons, the nucleotide at position 37 is usually not modified, except in Val(cmo^5^UAC), where it is modified to m^6^A37, and in Asp(gluQUC) and Glu(QUC), where it is modified to m^2^A37. With A36-ending anticodons, the modification at position 37 is usually ms^2^i^6^A37, except in one case Sec(UCA), where it is modified to just i^6^A37. Notably, with G36-ending anticodons, the modification at position 37 is m^1^G37 or m^2^A37, where the former is in Leu(cmo^5^UAG), Leu(GAG), Leu(CAG), Pro(mcmo^5^UGG), Pro(GGG), and Pro(CGG), and in Arg(CGG), while the latter is in His(QUC), Gln(cmnm^5^s^2^UUG), and in Arg(ICG). The nucleotide coupling between G36 and m^1^G37 has an enzymatic rationale, where the modification enzyme TrmD for m^1^G37 in bacteria indeed requires G36 as a recognition element to perform the modification reaction (Hou et al., 2017). This enzymatic rationale is likely to hold for other 36–37 nucleotide pairs, although experimental validation is necessary.

## A potential modification circuitry between tRNA positions 34 and 37

Given the importance of the modifications at both positions 34 and 37, we explore the possibility that some of them may have co-evolved with an inter-dependent relationship in a concept known as the “modification circuitry” (Han and Phizicky, 2018; Barraud and Tisne, 2019). Indeed, the presence of the m^1^G37 modification in *E. coli* Pro(mcmo^5^UGG) is the prerequisite for the methoxyl addition to convert cmo^5^U34 to mcmo^5^U34 (Masuda et al., 2018), while the presence of ms^2^i^6^A37 in *E. coli* Leu(C_m_AA) and Leu(cmnm^5^U_m_AA) is the prerequisite for 2′-*O*-methylation in C_m_34 and U_m_34, respectively (Zhou et al., 2015). Similarly, the formation of C_m_32 and C_m_34 in the ASL of yeast Phe(GAA) is the prerequisite to convert m^1^G37 to yW37 (wybutosine) (Guy et al., 2012). To further evaluate the concept of the modification circuitry in the *E. coli* modification pattern, we analyzed the co-existence of the modifications at positions 34 and 37. Notably, we emphasize that co-existence only provides an implicit for circuitry, which requires experimental validation.

The list of a potential modification circuitry is complex and idiosyncratic, indicating that it is strongly dependent on individual ASL sequences, as well as the presence of other modifications. Here, we provide a framework for the potential circuitry of modifications at positions 34 and 37. We note that the cmnm^5^ and mnm^5^U34 series of modifications are often associated with i^6^A37 and t^6^A37 series of modifications generated by one of the more complex enzymatic pathways (Figure 1B). For example, cmnm^5^U34 and mnm^5^U34 co-exist with ms^2^i^6^A37 in Leu(cmnm^5^U_m_AA) and with ct^6^A37 in Arg(mnm^5^UCU), respectively. However, addition of the 2-thio (s^2^) group to form cmnm^5^s^2^U34 or mnm^5^s^2^U34 simplifies the modification at position 37 to usually a single methyl group (Figure 1C). This is shown in the association of cmnm^5^s^2^U34 with m^2^A37 in Gln(cmnm^5^s^2^UUG) and the association of mnm^5^s^2^U34 with m^2^A37 in Glu(mnm^5^s^2^UUC). An exception is the association of mnm^5^s^2^U34 with ct^6^A37 in Lys(mnm^5^s^2^UUU), where both modifications are generated from a multi-enzyme pathway. In contrast, while the cmo^5^U34 modification is generally associated with the simple structure of m^1^G37 (Figure 1D), such as in Leu(cmo^5^UAG), the addition of one methyl group to form mcmo^5^U34 requires a more complex structure at position 37 (Figure 1E), such as ms^2^i^6^A37 in Ser(mcmo^5^UGA) and ct^6^A37 in Thr(mcmo^5^UGU). An exception is that the mcmo^5^U34 modification remains associated with m^1^G37 in Pro(mcmo^5^UGG).

In contrast, the pairing of Q34 is diverse. While it is paired with the simple structure of m^2^A37 in His(QUG) and in Asp(gluQUC) (Figure 1F), it is paired with the complex structure of ms^2^i^6^A37 in Tyr(QUA) and the complex structure of ct^6^A37 in Asn(QUU) (Figure 1G).

## Perspectives

Here we provide an easily accessible modification pattern of *E. coli* tRNAs that is integrated into the genetic code. In this modification pattern, each anticodon that reads the corresponding codon is shown with the modifications at positions 34 and 37. These modifications are likely selected during evolution to serve two functions – to facilitate accurate and diverse reading of each codon and to neutralize differences among various codon-anticodon pairings to ensure a uniform speed and quality of decoding across all codons, which is important for protein homeostasis (Ranjan and Rodnina, 2016). As these modifications are contained within the associated tRNA, which is charged with the specified amino acid for the codon, they constitute the necessary mechanism to facilitate interpretation of the genetic code. Despite the diverse structures of these modifications and their complex distribution in the genetic code, we have elucidated two principles for how each anticodon is associated with modifications to facilitate interpretation of the codon. These principles provide the basis to guide the users to search for more detailed information.

First, we find that modifications of complex structures are usually associated with AU-rich codons, while modifications of a simple structure or no modification are associated with GC-rich codons. Indeed, U34 is almost always modified with chemical structures that are synthesized by a multi-enzyme pathway and A34 is exclusively modified to I34, whereas C34 is not necessarily modified, while G34 is modified to Q34 when it is followed by a uridine on the 3′-side of the anticodon. At position 37, which stabilizes the stacking interaction with the nucleotide at position 36, the modification at 37 is usually a complex structure when the preceding nucleotide is U36 or A36. In contrast, the nucleotide at position 37 is not modified when the preceding nucleotide is C36, while the nucleotide at position 37 is modified to the simple structure of m^1^G37 when the preceding nucleotide is G36. In one of the most AU-rich cases, Lys(mnm^5^s^2^UUU) is modified with mnm^5^s^2^U34 and with ct^6^A37, both generated from a complex pathway.

Second, we suggest the possibility of a diverse, but potentially important, circuitry relationship between modifications at positions 34 and 37. Specifically, the addition of a simple chemical moiety at position 34 can change the profile of the modifications at position 37. This change is likely sequence-dependent to optimize each ASL to dock to the ribosome decoding site, which is structured by the 16S rRNA that is also extensively modified (Demirci et al., 2010; Kimura and Suzuki, 2010). While further experimental validation is necessary to validate the potential modification circuitry at positions 34 and 37 of each ASL, additional consideration of the circuitry should include the potential base pairing interactions at positions 31–39 and at 32–38, as they can also modulate the quality of the codon-anticodon pairing (Grosjean and Westhof, 2016).

Notably, several bacterial pathogens lack specific tRNA modifications, emphasizing that a more detailed study of these modifications will be important to human health. For example, our homology search suggests that *Pseudomonas aeruginosa* and *Acinetobacter baumannii* lack the gene for TmcA to synthesize ac^4^C34 on Met(CAU) and the gene for TrmM to synthesize m^6^A37 on Val(UAC). Additionally, our homology search suggests that *A. baumannii* lack the genes for CmoA and CmoB to synthesize cmo^5^U34, which would be required to expand wobble pairing in several tRNAs. This raises the question of how these bacteria species achieve the quality and diversity of anticodon-codon pairing to remain viable. Additionally, *Mycobacterium bovis* alters the level of cmo^5^U34 to adapt to hypoxia (Chionh et al., 2016) and *P. aeruginosa* alters the level of C_m_/U_m_/A_m_32 to adapt to oxidative stress (Jaroensuk et al., 2016), reinforcing the notion that tRNA modifications play an essential role in stress response of bacterial pathogens.

In summary, the development of the modification pattern for *E. coli* tRNAs provides an important reference that will facilitate researchers to investigate the origin and evolution of the genetic code, the interpretation of the code during protein synthesis, and the exploration of the code for bioengineering purposes. It will also provide a rewarding path to investigate the potentially even more complex landscape of modifications in higher eukaryotes.

## Data availability statement

The original contributions presented in the study are included in the article, further inquiries can be directed to the corresponding author.

## Author contributions

IM: Conceptualization, Investigation, Visualization, Writing – review & editing, Data curation. Y-MH: Conceptualization, Funding acquisition, Supervision, Validation, Visualization, Writing – original draft, Writing – review & editing, Project administration.
